# Renal Mucosa-Associated Lymphoid Tissue (MALT) Associated End-Stage Renal Disease in a Patient Presenting With Diarrhea

**DOI:** 10.7759/cureus.16158

**Published:** 2021-07-04

**Authors:** Faran S Polani, Fawwad Zaidi

**Affiliations:** 1 Division of Oncology, Department of Internal Medicine, Barnes-Jewish Hospital and The Alvin J. Siteman Comprehensive Cancer Center, Washington University School of Medicine, Saint Louis, USA; 2 Division of Oncology, Department of Internal Medicine, Simmons Cancer Center, Southern Illinois University School of Medicine, Springfield, USA

**Keywords:** marginal zone b cell lymphoma, renal lymphoma, end-stage renal disease (esrd), mucosa-associated lymphoid tissue (malt), anti-cd20 antibody

## Abstract

Extranodal marginal zone lymphoma (MZL) of mucosa-associated lymphoid tissue (MALT) is most commonly found in the GI tract. Other less common anatomical sites for MALT include the skin, intestine, salivary glands, lungs, and ocular adnexa. Isolated MALT of the kidney has only been sporadically reported. Most of the reported cases in the literature present with underlying renal mass and are generally diagnosed post nephrectomy. We present a case of a 73-year-old gentleman with biopsy-proven primary MALT of the kidney who presented with acute kidney injury (AKI) in the background of *Clostridium difficile (C. difficile)* colitis. However, our patient did not have a renal mass a renal biopsy was performed due to accelerated deterioration of renal function. Due to the inherent heterogeneity of the disease, it is challenging to have a unifying treatment strategy for MALT with treatment varying with the anatomical site. We also discuss current and prospective treatment strategies for MALT and marginal zone lymphoma in general.

## Introduction

Marginal zone lymphoma (MZL) is a low-grade B-cell non-Hodgkin’s lymphoma found in the external part of secondary lymphoid follicles, hence the name marginal zone lymphoma. According to the 2016 WHO classification of lymphoid neoplasia, MZL can be divided into nodal marginal zone lymphoma (NMZL) involving lymph nodes, splenic marginal zone lymphoma (SMZL) involving the splenic lymphoid tissue, and extranodal marginal zone lymphoma of mucosa-associated lymphoid tissue (MALT) [1]. The stomach is the most common anatomical site for MALT, followed by the skin, lungs, intestines, and ocular adnexa. The primary involvement of kidneys in MALT is rare and only sporadically reported [2]. MALT is usually attributed to chronic inflammation as a result of infection or autoimmune disorders. Here we present a case of a 73-year-old gentleman who presented with diarrhea, secondary to *Clostridium difficile (C. difficile)* infection. He developed end-stage renal disease (ESRD), prompting a kidney biopsy that led to a diagnosis of MALT. We also discuss the heterogeneity of MZL and various treatment strategies recently approved or in ongoing clinical trials.

## Case presentation

A 73-year-old Caucasian male presented to the hospital for acute renal failure secondary to diarrhea and dehydration. The patient was found to have C. difficile infection which was treated with oral vancomycin and Flagyl. He had a past medical history of diabetes on metformin, hypertension, and hyperlipidemia, all diagnosed in the last six months before presentation. Family history was significant for coronary artery disease (CAD) in his brother at an unknown age. There was no family history of hematological malignancy or autoimmune disorders. There was no history of chronic non-steroidal anti-inflammatory drug (NSAID) use. The patient denied any history of smoking or recreational drug use. He used to drink alcohol occasionally and worked in a coffee factory all his life. There was no history of radiation exposure.

Upon presentation, the patient's basic metabolic panel showed creatinine of 15, sodium of 140, potassium of 5.6, chloride of 106, blood urea nitrogen of 95, calcium of 9.2, and bicarbonate of 20. Complete blood count showed a WBC count of 8.3X10^3^/μL, neutrophils 74%, hemoglobin 12.7 g/dL, and platelet count of 253 X10^3^/μL. Renal biopsy was done which showed acute tubular injury with an increased number of calcium oxalate crystals and arteriolosclerosis. It also showed low-grade large B-cell lymphoma immunophenotypically most consistent with extranodal marginal zone lymphoma of MALT, involving approximately 10% of the kidney tissue. Immunohistochemistry of the kidney tissue showed CD 19, CD20 positive B cells, that were negative for CD5, CD10, CD23, CD200, CD38, and MYD 88. The Ki-67 proliferative index was low.

CT of the abdomen and pelvis showed no bowel obstruction or appendicitis or acute inflammatory change. Non- obstructing right renal lithiasis was found. No hydronephrosis or obstructive uropathy was noted. No lymphadenopathy was noted on the CT. Autoimmune workup including antinuclear antibodies (ANA), anti-double-stranded DNA, and antineutrophil cytoplasmic antibodies (ANCA) was negative. HIV and hepatitis panel, including hepatitis B and hepatitis C, were nonreactive.

**Figure 1:**
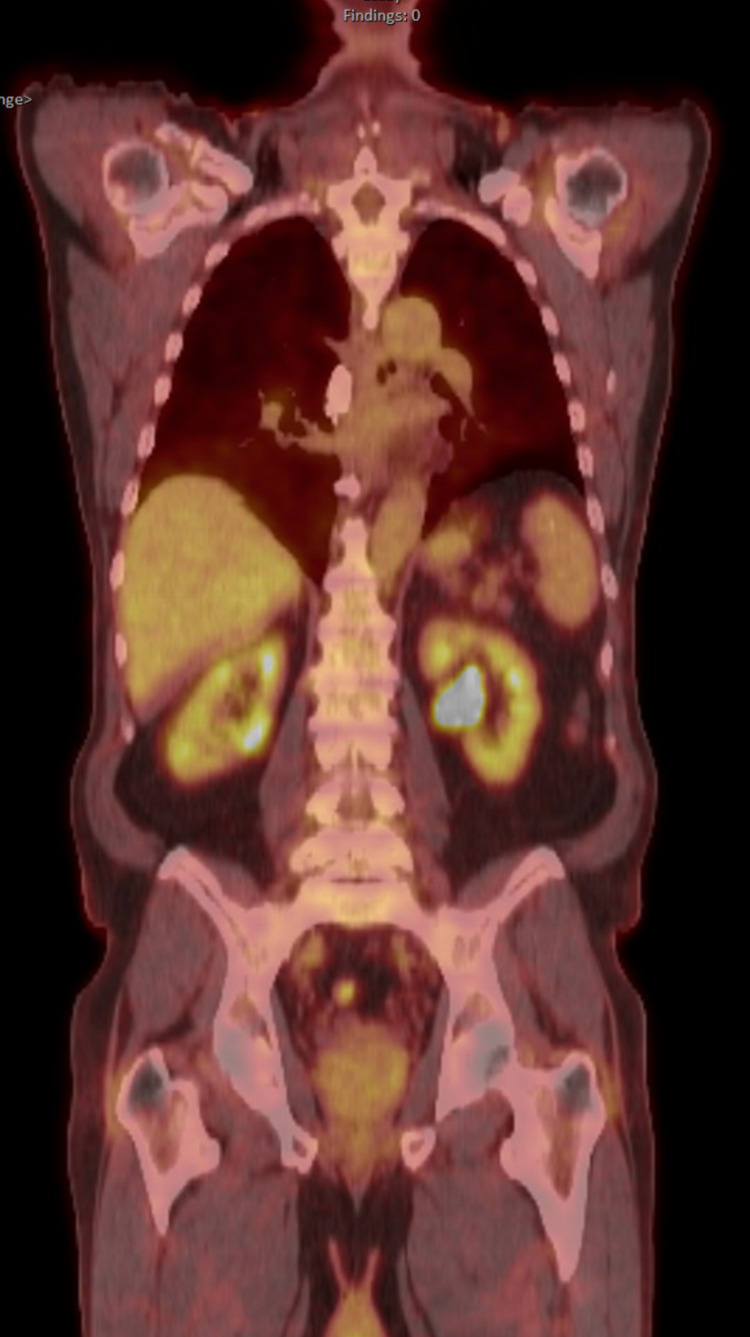
PET CT Uniform activity in renal parenchyma with no other significant hyper-metabolic activity or lymphadenopathy

A positron emission tomography (PET) scan (Figure 1) showed uniform activity in renal parenchyma with no other significant hyper-metabolic activity or lymphadenopathy.

Bone marrow biopsy showed normocellular marrow involved by low-grade B-cell non-Hodgkin lymphoma consistent with marginal zone lymphoma, approximately 10%-15% involvement. Flow cytometric analysis of the bone marrow aspirate showed approximately 33% of lymphocytes that were monotypic B-cells, expressing CD19, CD20, CD22, CD45, and showed lambda light chain restriction. The B-cells were negative for CD5, CD10, CD23, CD200, and CD38.

The patient was started on dialysis, and he remained on intermittent hemodialysis three times a week for two months. However, his renal function improved without any treatments and interventions for his MZL. The patient eventually came off dialysis. Since the patient now remains completely asymptomatic, he did not receive any treatment for his MZL. The patient will follow up in a lymphoma clinic in three months with a repeat CT abdomen pelvis for close monitoring of his disease.

## Discussion

MALT is associated with chronic inflammation secondary to infections like *Helicobacter Pylori (H. Pylori)* in gastric MALT or autoimmune disorders like Sjogren’s syndromes in MALT of salivary glands. The predisposition factor for isolated MALT of kidneys is not well-established. There are only a handful of cases of MALT involving kidneys reported in the literature [2, 3]. Among them, concurrent renal actinomyces infection, Epstein-Barr virus (EBV) infection, sarcoidosis, immunoglobulin A (IgA) nephropathy, and membranoproliferative glomerulonephritis (MPGN) have been reported [4]. Only in a few cases, the diagnosis is made on renal biopsy. Due to the disease's indolent nature, most of the diagnosis of localized MALT of the kidneys is made post nephrectomy, usually done for suspected renal cell carcinoma. In such cases, the patients are usually not treated with chemotherapy or radiation and are managed expectantly.

Most of the cases of MZL are diagnosed at Ann Arbor Stage I E. Only 20% of the cases have systemic BM and peripheral lymph node involvement at the time of diagnosis. MALT is an indolent disease and tends to remain isolated at the site of origin. The five-year survival rate of MALT patients, depending on the anatomical site, ranged from 69.1 to 87.9% [5]. The risk of advancement to more aggressive large B-cell lymphoma is roughly 10%[6].

Due to the underlying heterogeneity of the disease, it is challenging to have a unifying treatment strategy for MALT patients. The treatment modality of MALT primarily depends on the anatomical site of origin. Gastric MALT in patients positive for *H. pylori* is treated with* H. pylori* eradication followed by endoscopic surveillance. In patients with H.Pylori negative MALT, radiotherapy is recommended in the early stage of the disease. In anatomical sites where radiotherapy is not an option, surgical management or single-agent anti-CD-20 therapy like rituximab is an established strategy. Bendamustine plus rituximab combination has also been used as first-line therapy in MZL patients with favorable outcomes and without overt toxicities [7]. Patients with disease that has progressed on rituximab or relapsed can be treated with an increasing number of non-chemotherapy options or combination therapies. The B-cell receptor signaling pathway is an important component of lymphomagenesis; ibrutinib is Bruton’s tyrosine kinase inhibitor that inhibits this pathway. Ibrutinib has been approved by the FDA as a single-agent oral treatment specifically for relapsed/refractory (R/R) MZL [8]. A phase III AUGMENT trial involving 358 patients with R/R follicular lymphoma and MZL showed improved efficacy of rituximab when used in combination with lenalidomide, an immune-modulating drug that binds to the cereblon E3 ubiquitin ligase complex. Patients receiving the combination therapy had progression-free survival of 39.4 months (95% CI, 22.9 months to not reached) versus 14.1 months (95% CI, 11.4 to 16.7 months) for patients receiving placebo plus rituximab (NCT01938001) [9]. The FDA has recently approved a combination of rituximab and lenalidomide in R/R MZL largely based on the results of this trial. A number of prospective clinical trials are currently underway using different combinations of anti-CD20 antibodies and phosphoinositide 3-kinase (PI3K) inhibitors like copanlisib, buparlisib, umbralisib, duvelisib in patients with R/R MZL.

## Conclusions

We report the case of a 73-year-old gentleman who presented with acute kidney injury (AKI) and *C. difficile* colitis later found to have renal MALT on renal biopsy carried out due to accelerated decline in renal function. Renal MALT is very rare and most of the cases reported present with a renal mass and associated symptoms, with diagnosis mostly done post nephrectomy. In our case, the patient did not have a renal mass. This emphasizes the importance of a renal biopsy in patients with a quick decline of renal function. Our patient is not dialysis-dependent anymore. His MALT is limited to his kidneys and bone marrow with no nodal involvement. He is currently being managed expectantly. In symptomatic patients, MZL is either treated with radiation, surgery, or a combination of chemotherapy and immunotherapy, with the stage and anatomical location of the disease dictating the therapeutic strategy. Immunotherapies like ibrutinib and lenalidomide have recently been approved by the FDA specifically for R/R MZL.
